# PROTOCOL: Improving access to health and social services for individuals experiencing, or at risk of experiencing, homelessness

**DOI:** 10.1002/cl2.1118

**Published:** 2020-11-18

**Authors:** Sarah Miller, Ciara Keenan, Jennifer Hanratty, Jayne Hamilton, Christopher Coughlan, Peter Mackie, Suzanne Fitzpatrick, Alan Maddock

**Affiliations:** ^1^ Centre for Evidence and Social Innovation, Campbell UK & Ireland Queen's University Belfast Belfast UK; ^2^ School of Geography and Planning Cardiff University Cardiff UK; ^3^ Institute for Social Policy, Housing, Environment and Real Estate (I‐SPHERE) Heriot‐Watt University Edinburgh UK; ^4^ School of Social Sciences, Education and Social Work Queen's University Belfast Belfast UK

## 1 PLAIN LANGUAGE SUMMARY

The title should be in headline style summarising the main findings of the review, for example, “enforcing conditions makes cash transfers more effective in increasing enrolments” and “detention of asylum seekers has adverse effects on mental health”. The title for empty reviews can state that there is no evidence, for example, “there is no rigorous evidence on the effectiveness of refugee resettlement programs”. Titles can also reflect the size of the effects or the quality of the evidence, for example, “Limited evidence and limited effects of advocacy to reduce intimate partner violence”.

### 1.1 The review in brief

A short summary of the main findings of the review. This section may be no more than one sentence, and should not exceed 50 words. For example, “custodial sentences are no better than non‐custodial sentences in reducing re‐offending”.

Selective outcome reporting is to be avoided. So reviews with several primary outcomes will require a longer review in brief section, for example, “Intensive advocacy may improve everyday life for women in domestic violence shelters/refuges and reduce physical abuse. There is no clear evidence that intensive advocacy reduces sexual, emotional, or overall abuse, or that it benefits women's mental health. It is unclear whether brief advocacy is effective”.

### 1.2 What is this review about?

This section should include:

- A “problem statement” of the issue being addressed. For example, “half of all crime takes place in small, localised areas, or hot spots”; and “forests are an important resource for managing climate change because they store carbon, which helps mitigates the effect of carbon emissions. However, the amount of forest cover, particularly in low‐ and middle‐income countries (LMICs), is declining. Deforestation is responsible for 10–17% of global carbon emissions”.
- A clear description of the intervention being assessed. For example, “Payment for environmental services are voluntary contracts to supply a well‐defined environmental service in exchange for payment. For the purposes of this review, the service must involve the maintenance or rehabilitation of natural forests”.
- The outcomes included in the review. For example “this review looked at whether custodial and alternative noncustodial sanctions have different effects on the rates of re‐offending”.
- Optional: the policy question being addressed. For example, “the review considers evidence regarding the debate about whether PESs should also aim to reduce poverty, or whether doing so would undermine conservation efforts”.

### 1.3 What is the aim of this review?

People do not always understand that the results of a plain language summary come from a systematic review rather than a single study. Some also wrongly assume that the review authors have carried out the studies themselves. A text box should be included on the first page stating what the review studied, and how many studies were included.

For example: This Campbell systematic review examines the effects custodial sentences on reoffending, compared to the effects of noncustodial sentences. The review summarises evidence from fourteen high‐quality studies, including three randomised controlled trials and two natural experiments.

### 1.4 What are the main findings of this review?

#### 1.4.1 First subheading: “What studies are included?”

A brief description of the number of included studies and key characteristics (e.g., study design and region or country). For example, “this review includes studies that evaluate the effects of custodial and non‐custodial sanctions on reoffending. A total of 38 studies were identified. However, only 14 of these were assessed to be of sufficient methodological quality to be included in the final analysis. The studies spanned the period from 1961 to 2013 and were mostly carried out in the USA, Europe and Australia”.

*Optional*: add a statement about the quality of the evidence. For example, “the studies all had some important methodological weaknesses. None of the included studies used experimental designs (random assignment)”.

*Additional subheadings* state the question being answered in that section, for example, “Does focusing crime prevention efforts on crime hot spots reduce crime?” and “What factors affect how well PES programmes work?”

These sub‐sections give a short summary of the review evidence to answer that question. Present the results consistently, using similar words and expressions for similar levels of effect (see Appendix 1 for suggested wordings). Ensure that the results are reported consistently between the plain language summary and the main text of the review, including the abstract, results and summary of main results. For example, “Yes. There is an overall reduction in crime and disorder when hot spots policing interventions are implemented. The largest reductions are in drug offences, violent crime and disorder offences, with smaller reductions in property crime”.

*Notes*:

(1) The findings are presently directly, and in the present tense. So do not write “the authors found” or “the review found”.
(2) Avoid selective reporting. The results for each main outcome must be presented in the section called “What are the main findings?” (or a variation specific to the review such as “Does focusing crime prevention efforts on crime hot spots reduce crime?”). If you found no data on an important outcome, you must present the outcome anyway, but explain that no data were found.

Using qualitative statements when presenting the effects of the intervention: You may be able to increase the accessibility of the review by avoiding numbers and using qualitative statements to present the results. By “qualitative statements” we mean an expression of your results in plain language, using similar words and expressions for similar levels of effect. Qualitative statements about effect are difficult to get right. It is easy to cause confusion and misinterpretation by using words inconsistently or statements such as “a high likelihood of somewhat small but possibly important effects”.

#### 1.4.2 Optional subheading: How has this intervention worked?

Present here the evidence relating to the main assumptions and links in the theory of change for the intervention(s) being assessed. The findings with respect to intermediate outcomes can be reported here.

### 1.5 What do the findings of this review mean?

Include here the main policy relevant findings and their implications for policy and further research. Reviews do not make policy recommendations. Include also implications for research.

### 1.6 How up‐to‐date is this review?

State here when the review authors searched for the included studies: “The review authors searched for studies up to 2015. This Campbell Systematic Review was published in January 2017”.

## 2 BACKGROUND

### 2.1 The problem, condition or issue

Homelessness is a multifaceted issue with outcomes that are as complex and unique as the individual who is experiencing life without stable housing. Those people who are currently experiencing homelessness have a much greater risk of poorer physical and mental health than the general population (Homeless Link), and so the requirement to access health and social care (HSC) services is increased.

Homelessness affects individuals who are experiencing life without safe, adequate, or stable housing. Conceived in this way, homelessness not only describes those individuals who are visibly homeless and living on the street, but also those precariously housed individuals who; stay in emergency accommodation, sleep in crowded or inadequate housing, and those who are not safe in their living environment. FEANTSA further classify individuals experiencing homelessness as those who are roofless, those who are houseless and those who experience insecure or inadequate housing (Feantsa, 2005).

Accessing HSC services while homeless is problematic for several reasons. First, there are many countries in the world without a free health care system and individuals experiencing homelessness may need to prioritise food and temporary shelter over their basic HSC needs (Hoshide, Manog, Noh, & Omori, 2011). Second, there can be difficulties associated with registering for HSC services due to practical issues such as the requirement of documentation including; health insurance, personal identification, national insurance/social security numbers, or current address details (Feldman et al., 2017). Third, issues with the location of the HSC services may be an additional barrier to those without access to transport (Syed, Gerber, & Sharp, 2013). Fourth, it may be that there is a lack of suitable HSC services to meet an individual's needs, or there may be a waiting list that delays a person's access to the service they require (Hudson et al., 2010). Fifth, the individual may be someone who has multiple HSC needs. The range and complexity of these needs, along with the potentially chaotic nature of the individual's life as a result may make it particularly difficult for the person experiencing homelessness to access all the services that they require without HSC support (Moore, Manias, & Gerdtz Marie, 2011). This is due to the fact that meeting these needs may entail managing a range of professional appointments in a variety of different HSC settings. Sixth, people experiencing homelessness, who may be dealing with one or more HSC needs, may live in, or be placed in living environments, for example, hostel accommodation, which may not be suitable to their recovery pathways. The increased stress that these living arrangements may cause may reduce the individual's HSC outcomes, for example, a person with an underlying mental health issue may experience increased anxiety and/or depression. A person with an addiction issue may also relapse into alcohol or drug addiction due to this increased environmental stress coupled with the decreased difficulty in procuring the substances that they may have an issue with. An exacerbation of one or both of these HSC needs may lead to a decreased capacity on the part of the person who is experiencing homelessness to access relevant HSC services or remain compliant with HSC services that they were engaging with (Padgett, Tiderington, Tran Smith, Derejko, & Henwood, 2016). Finally, people experiencing homelessness will experience high levels of prejudice and discrimination (Weng & Clark, 2018) and so fear of these pervasive attitudes and behaviours, coupled with low confidence and self‐esteem, may inhibit access to HSC services for those who require them most.

Previous research has demonstrated that gender may play an important role in HSC needs and how services are used. For example, males were more likely to report substance abuse (Berdahl, Hoyt, & Whitbeck, 2005), females were more likely to have suffered sexual harassment and assault (Ensign & Panke, 2002), and are much more likely to seek out and use health care services (Ensign, 2003; Piliavin, Westerfelt, Wong, & Afflerbach, 1994).

### 2.2 The intervention

Individuals experiencing homelessness often have various comorbidities requiring support from a range of HSC services. These services may be provided by professionals such as general practitioners, psychiatrists, psychologists, social workers, community nurses, psychotherapists, occupational therapists, hospital staff, dentists, pharmacists, and staff working in housing, employment or education services. As outlined previously, a person experiencing homelessness may face seemingly impenetrable barriers to accessing these services and professionals.

To improve access to HSC services, interventions must address the barriers which exist. This systematic review will include all relevant studies which assess the effectiveness of interventions which aim to improve the accessibility of HSC services for individuals experiencing, or at risk of experiencing homelessness. Here we will focus on interventions which change something about how, where, or to whom the service is delivered or where the services actively seek to remove barriers to access for this population. Although related, this systematic review is not concerned with those studies which assess the effectiveness of the HSC services themselves. We will only include interventions that seek to change something about the service, and how it is delivered, to increase its accessibility. We will exclude interventions that only seek to change the service users' behaviour or provide support for a person to access an existing service. Service level change must be a component of the intervention. For example, many case management programmes will assign a case manager to work with individuals to help them to navigate the complex landscape of services and entitlements in their local area. This approach does not typically involve the services themselves making changes to improve accessibility.

### 2.3 How the intervention might work

**Figure 1:**
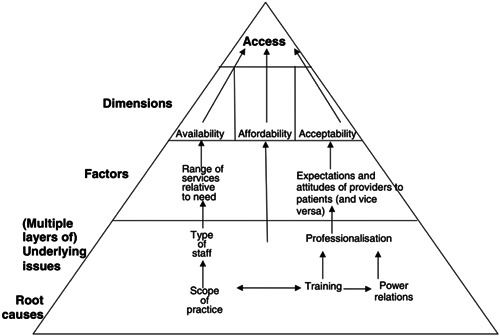
Access evaluation framework as presented in McIntyre et al. (2009)

McIntyre et al. (2009) have created a conceptual framework (Figure 1) to capture the precise meaning of access to health care in low‐ and middle‐income countries. The three dimensions identified by the study authors are availability, affordability and acceptability. This research provides a framework for this review to both categorise the studies and understand the barriers and opportunities that may influence access to HSC services for individuals experiencing homelessness. These dimensions are described in more detail below.

#### 2.3.1 Availability

Interventions included in this category are those that address whether the appropriate HSC services are delivered in the right location and at the right time to effectively meet the needs of the population. These interventions may address barriers such as:

- The physical location of HSC services in relation to the service user (e.g., are clinics which deal with human immunodeficiency virus [HIV] related illness situated in places where there is a high proportion of homeless individuals with HIV?).
- System factors such as the opening hours of services and how appointment systems are managed (e.g., ensuring that service hours are also available outside working hours to allow employed individuals to avail of the services they require).
- Relationship between what the service provider has to offer and what the service user requires (e.g., a service provider which seeks to provide care in a holistic way so that the service user is not required to wait for multiple referrals from various providers. One example might be the integration of an outpatient treatment centre within a primary care setting).

#### 2.3.2 Affordability

Interventions in this category are those which address the gap between the costs of the service against the person's ability to pay. Most interventions included in this category focus on the provision of public funding, vouchers, or charitable donations to enable a person to access the services they require. These interventions to address affordability of HSC services work to remove barriers associated with the following factors:

- The price of the service at the point of delivery (e.g., a scheme which provides free medical care to the service user).
- Direct costs (McIntyre et al., 2009) describe direct costs using examples such as transportation, special diets, child care (e.g., providing free insulin to individuals who are diabetic).
- Indirect costs (McIntyre et al., 2009) describe indirect costs using examples such as lost income or productivity while travelling to and from, and waiting to be seen by, a health care provider. For example, if a person has a mental illness and is employed, they may be unable to access a service due to the time requirements. A relevant intervention may provide monetary cover for the loss of income while they avail of the necessary service.

#### 2.3.3 Acceptability

This category contains interventions which focus on preparedness of different HSC professional groupings, either individually or within multidisciplinary teams, to address the HSC needs of specific individuals. Different HSC professional groupings tend to have differing perspectives on the underlying causes of specific issues and how best to support these needs. This is based on the training that the individual professions receive and their professional socialisation. These differing perspectives can have a profound effect on the ability of people who are experiencing homelessness to access HSC services. HSC services which operate out of a psychosocial model are likely to promote access for people who are experiencing homelessness due to their person‐ centred nature and wider assessment criteria. HSC services which would adhereadhering to the medical model would likely reduce access to services for people who are experiencing homelessness, unless their needs fit within its more stringent diagnostic criteria. Interventions here may focus primarily on the attitudes and expectations each group will have of the other, and how addressing these pervasive cognitive factors may improve access to services.

Some examples of these include:

- Provider expectations (e.g., a provider may offer extended and additional services for those who adhere to the prescribed treatment).
- User expectations (e.g., the desire to be listened to and respected when describing service needs. Perhaps enhancing autonomy of care by creating a comprehensive care plan between the service provider and user).
- Improving cooperation between service providers to meet the service usersuser's expectations (e.g., minimising the burden placed on individuals experiencing homelessness, by creating a smoother referral process and improving communication between statutory, community and voluntary organisations offering HSC services).

Facilitating access to HSC services helps those individuals experiencing homelessness lead more independent, healthy and happy lives while retaining autonomy over their HSC choices. When a service that someone requires and is entitled to is inaccessible, they cannot receive the treatment and support available, meaning it has failed to meet their needs.

Considerations for improving access to HSC services include the physical environment of the service, staff levels of awareness, empathy and understanding, eligibility criteria for accessing the service and the physical location where the service is delivered.

### 2.4 Why it is important to do this review

The United Kingdom's National Health Service (NHS) recognises the need to improve access to services especially to underprivileged groups as outlined in principle one of the NHS constitution.

“The NHS provides a comprehensive service, available to all…it has a duty to each and every individual that it serves and must respect their human rights. At the same time, it has a wider social duty to promote equality through the services it provides and to pay particular attention to groups or sections of society where improvements in health and life expectancy are not keeping pace with the rest of the population”.

To ensure that policymakers and practitioners' avail of the most robust and rigorous evidence to date, there is a significant need to identify and combine all relevant interventions which aim to improve accessibility of HSC services for individuals experiencing, or at risk of experiencing homelessness. Through the proposed evidence synthesis, we can inform key stakeholders about where existing studies have been conducted and the quality of these. This will allow funders to see where research gaps exist and where there is already a saturation of available evidence. Through meta‐analysis we will explore whether certain subgroups of participants access services in different ways which will help understand the barriers that exist for various populations of people experiencing homelessness.

This systematic review will be based on evidence already identified in two existing evidence and gap maps (EGMs) commissioned by the Centre for Homelessness Impact (CHI) and built by White, Saran, Teixeira, Fitzpatrick, and Portes (2018). The EGMs present studies on the effectiveness and implementation of interventions aimed at people experiencing, or at risk of experiencing, homelessness. The EGMs were constructed using a comprehensive search strategy including a search of Campbell, PROSPERO and Cochrane databases. The EGMs identified various systematic reviews which assess the effectiveness of interventions to improve both physical and mental health in homeless populations (Hwang, Tolomiczenko, Kouyoumdjian, & Garner, 2005; Speirs, Johnson and Jirojwong, 2013; Thomas, Gray and McGinty, 2011) and reducing homelessness (Fitzpatrick‐Lewis et al., 2011; Munthe‐Kaas, Berg and Blaasvær, 2018) but fewer focus on those interventions which seek to improve access to HSC services. The author will outline those systematic reviews which synthesise the literature around interventions to improve access to HSC services and how they are different from the proposed review.

#### 2.4.1 Restricted by intervention

Three reviews have included only those interventions which use social networking sites to improve access to HIV prevention services. First, a systematic review by Capurro et al. (2014) identified 73 studies. However, as they focussed on a general population of participants described as “difficult to reach” only two studies which focussed on homeless youth were included (Rice, Tulbert, Cederbaum, Barman Adhikari, & Milburn, 2012; Young & Rice, 2011). Similarly, a second systematic review (Lim, Wright, Carrotte, & Pedrana, 2016) of 47 studies found only one which included a homeless population (Rice et al., 2012). Thirdly, in a systematic review which located 58 social network based interventions (Ghosh et al., 2017) five were on homeless men and youths.

Another systematic review identified by the map did centre on a homeless population (McInnes, Li, & Hogan, 2013). However, it focussed on their access to information technologies such as mobile phones and the internet. The authors do conclude that this access to technology will improve access to HSC, but this was not tested within the review.

#### 2.4.2 Restricted by population

Three reviews have included only specific subsets of the homeless population. First, a review by Hudson et al. (2016) included nine qualitative studies which focussed on access to services of those individuals requiring palliative care, while another systematic review of 62 studies (Brown, Rice Simon, Rickwood Debra, & Parker Alexandra, 2016) focussed on those individuals requiring mental‐health care only. Third, a systematic review of 12 studies conducted within the European Union (de Vries et al., 2017) focussed on access to diagnostic and treatment services for tuberculosis patients.

#### 2.4.3 Restricted by outcome

Finally, although a systematic review of five studies exists which has similar objectives to the current review (Health Quality Ontario), one of the inclusion criteria is more limited than the current review. Authors retrieved only those interventions that would improve access to a primary care provider (a physician, a nurse or a nurse practitioner). This review will be wider in scope and include interventions which seek to improve accessibility of any HSC services, not just primary care.

## 3 OBJECTIVES

1. What is the effect of interventions to improve accessibility of health and social services on outcomes for individuals experiencing or at risk of experiencing homelessness?
2. Who do these interventions work best for?
  a. Complexity of needs.
  b. Age.
  c. The presence of dependent children, in other words families compared to single individuals.
  d. Gender.
  e. Type of homelessness experienced.
3. What implementation and process factors impact intervention delivery?
4. Is implementation fidelity related to the effectiveness of the intervention?

## 4 METHODS

### 4.1 Criteria for considering studies for this review

#### 4.1.1 Types of studies

We will include all study designs where a comparison group is used. This includes randomised controlled trials, quasiexperimental designs, matched comparisons and other study designs that attempt to isolate the impact of the intervention on homelessness using appropriate statistical modelling techniques. These designs are chosen as the use of a control group helps ensure that changes observed in treatment group participants are due to effects of the intervention, and not attributable to other factors. Any study which includes only one group pretest/posttest or in which a treatment group was compared to another treatment group will not be eligible for inclusion.

As randomised controlled trials are accepted as more rigorous than nonrandomised studies, the potential impact of nonrandom study design on effect sizes will be explored as part of the analysis of heterogeneity.

Studies must include an inactive comparison condition that could include;

- No treatment.
- Treatment as usual where there is no service level changes to accessibility. Details of what this consists of will be extracted.
- Waiting list where service providers or service users are randomly assigned to receive the intervention at a later date. Details of what happens to waitlisted participants will be extracted.
- Attention control, where participants receive some contact from researchers but both participants and researchers are aware that this is not an active intervention.
- Placebo where participants perceive that they are receiving an active intervention but the researchers regard the treatment as inactive.

Studies with no control or comparison group, unmatched controls or national comparisons with no attempt to control for relevant covariates will not be included. Case studies, opinion pieces or editorials will not be included.

#### 4.1.2 Types of participants

This systematic review on accessibility of HSC services will focus on all individuals who are currently experiencing, or at risk of experiencing homelessness irrespective of age or gender. The included studies will include populations from high‐income countries. Homelessness is defined as those individuals who are sleeping “rough” (sometimes defined as street homeless), those in temporary accommodation (such as shelters and hostels), those in insecure accommodation (such as those facing eviction or in abusive or unsafe environments), and those in inadequate accommodation (environments which are unhygienic and/or overcrowded).

#### 4.1.3 Types of interventions

Interventions that will be included within this systematic review will be those with an explicit objective of improving accessibility of HSC services, we are not concerned with the effectiveness of the services themselves. HSC services will vary immensely according to factors such as resources available in each jurisdiction and/or the specific needs of the individual experiencing homelessness. Some examples of interventions may include:

- Those which seek to improve accessibility of a GP or nurse,
- Interventions which seek to improve collaboration between statutory, community and voluntary organisations offering HSC services in order to improve accessibility for people who are homeless or at risk of homelessness
- Those which improve the timeliness of access to all HSC services,
- Interventions which seek to educate HSC professionals on improving accessibility for individuals experiencing, or at risk of experiencing, homelessness
- Those interventions which adapt methods of communication and how information is presented to service users.

Comparison conditions will include services as usual or alternative services/intervention.

#### 4.1.4 Types of outcome measures

Briefly describe the types of outcome measures that will be included and excluded.

##### Primary outcomes

This review will primarily address how interventions can improve accessibility of HSC services for those individuals experiencing, or at risk of experiencing, homelessness. “Access” may be defined, measured and reported in different ways and may include descriptions such as: frequency of clients' contact with the service, uptake of support services, attendance of mental health programmes or utilisation of other health services. We will include all measures that report access to any health or social care service.

##### Secondary outcomes

Secondary outcomes include any other outcomes reported by studies that fall within the domains of the effectiveness EGM, which are:

- Community support for individual needs.
- Crime/criminalisation
- Employment and income
- Capabilities and wellbeing
- Cost effectiveness

We will also pay attention to implementation and acceptability of interventions and will include an analysis of attrition rates or “dropout” from interventions.

##### Duration of follow‐up

It is anticipated that the included interventions will report effects at multiple follow‐up periods after implementation of the intervention. In instances where this is the case, data relating to multiple points of follow up will be extracted in their entirety. This will allow us to conduct analysis on effect sizes related to similar time points and when outcomes are similar across various timepoints then an average effect size will be calculated to estimate effectiveness.

##### Types of settings

Settings where these interventions take place may be varied and can include community‐based settings, vocational settings, treatment centres, clinical settings and the individual's temporary accommodation.

### 4.2 Search methods for identification of studies

This systematic review will be based on evidence already identified in two existing EGMs commissioned by the CHI and built by White, Saran et al. (2018). The EGMs present studies on the effectiveness and implementation of interventions aimed at people experiencing, or at risk of experiencing, homelessness in high income countries. The maps used a comprehensive three stage search and mapping process. Stage one was to map the included studies in an existing Campbell review on homelessness (Munthe‐Kaas et al., 2018), stage two was a comprehensive search of 17 academic databases, three EGM databases and eight systematic review databases for primary studies and systematic reviews. Finally stage three included web searches for grey literature, scanning reference lists of included studies and consultation with experts to identify additional literature. Sample search terms can be found in the protocol (White, Saran et al., 2018).

We will not undertake any additional searching. However, if in the course of contacting authors for additional information or data necessary for conducting analysis and risk or bias assessments, authors provide us with additional eligible studies these would be included.

### 4.3 Data collection and analysis

#### 4.3.1 Description of methods used in primary research

Trials measuring the effectiveness of interventions to improve accessibility of HSC services against a control group or well matched comparison group will be included. An example of a study which is potentially relevant for inclusion in this review is The Keeping Home study (Appel, Tsemberis, Joseph, Stefancic, & Lambert‐Wacey, 2012). This study uses the Housing First approach, which Pathways to Housing, Inc. originated, to address the needs of homeless, seriously mentally ill substance abusers. Specifically, Keeping Home secures market‐rate, scattered site apartments for seriously mentally ill methadone maintenance treatment patients and then, through in vivo assertive community treatment supports (i.e., psychiatric, nursing, vocational, social and peer), addresses patients' service needs. The comparison group in this study receive Standard Care in the form of available housing and support services.

#### 4.3.2 Criteria for determination of independent findings

It is important to ensure that the effects of an individual intervention are only counted once and the following conventions will therefore apply.

Where there are multiple measures reported for the same outcome, we will use robust variance estimation to adjust for effect size dependency (Hedges, Tipton, & Johnson, 2010). The correction for small samples (Tipton & Pustejovsky, 2015) will be implemented when necessary. The exception will be any treatment inherent measures of the outcome of interest, these measurements will be discarded as they risk overestimating the treatment effect.

Where the same outcome construct is measured but across multiple time domains, such as through the collection of both post‐test and further follow‐up data, the analysis will conducted and reported separately for different time points (see above).

Studies comparing multiple treatment and control arms will be discussed with the full author team to decide if eligible intervention arms are similar enough to combine and compare as if they are one intervention group. If there are sufficient eligible studies reporting multiple and dependent effect sizes (i.e., occurring in more than 20 eligible studies) then robust variance estimation will be employed. This technique calculates the variance between effect sizes to give the variable of interest a quantifiable standard error. It has been shown to calculate correct results with a minimum of 20–30 individual studies (Hedges et al., 2010) although it performs better with an increased quantity of studies.

In the case of multiple cohorts appearing in one study we will calculate a simple average, as described above, for the omnibus meta‐analysis. If different cohorts in a study fall into different subgroups then they will be considered separately in subgroup analysis but no overall summary of effect will be calculated combining subgroups in those cases. If there are sufficient eligible studies reporting multiple and dependent effect sizes (i.e., occurring in more than 20 eligible studies) then robust variance estimation will be employed. This technique calculates the variance between effect sizes to give the variable of interest a quantifiable standard error. It has been shown to calculate correct results with a minimum of 20–30 individual studies (Hedges et al., 2010) although it performs better with an increased quantity of studies.

#### 4.3.3 Selection of studies

The studies contained within the exiting EGMs will be screened against the inclusion criteria for eligibility by two independent screeners.

#### 4.3.4 Data extraction and management

Once eligible studies have been found, we will undertake dual data extraction, where two authors will both complete data extraction and risk of bias assessments independently for each study. Coding will be carried out by trained researchers. Any discrepancies in screening or coding will be discussed with senior authors until a consensus is reached.

#### 4.3.5 Details of study coding categories

A coding framework has been developed and piloted prior to undertaking data extraction for all included studies using EPPI Reviewer software (see Summary of findings Tables). At a minimum we will extract the following data: publication details, intervention details including setting, dosage and implementation, delivery personnel, descriptions of the outcomes of interest including instruments used to measure, design and type of trial, sample size of treatment and control groups, data required to calculate Hedge's *g* effect sizes, quality assessment. We will also extract more detailed information on the interventions such as: duration and intensity of the programme, timing of delivery, key programme components (as described by study authors), theory of change. Alongside extracting data on programme components, descriptive information for each of the studies will be extracted and coded to allow for sensitivity and subgroup analysis. This will include information regarding:

- Setting, which type of institutional setting(s) are study participants transitioning from?
- The study characteristics in relation to: design, sample sizes, measures and attrition rates, who funded the study and potential conflicts of interest.
- Demographic variables relating to the participants including age, complexity of needs, dependent children, and other relevant population characteristics.

Quantitative data will be extracted to allow for calculation of effect sizes (such as mean change scores and standard error or pre and post means and standard deviations or binary 2 × 2 tables). Data will be extracted for the intervention and control group on the relevant outcomes measured in order to assess the intervention effects.

#### 4.3.6 Assessment of risk of bias in included studies

Assessment of methodological quality and potential for bias will be conducted using the second version of the Cochrane Risk of Bias tool for Randomised controlled trials (Higgins & Green, 2016). Non‐randomised studies will be coded using the ROBINS‐ I tool (Sterne et al., 2016).

#### 4.3.7 Measures of treatment effect

It is anticipated that most outcomes reported will be based upon continuous variables and so the main effect size metric to be used for the purposes of the meta‐analyses will be the standardised mean difference (SMD), with its 95% confidence interval.

Within this, Hedges' *g* will be used to correct for any small sample bias. SMD will be calculated from means and standard deviations in the first instance, however, if a study does not provide this raw data, authors will be contacted, and this information will be requested. Failing this, many papers have been published to assist authors in calculating the SMD from primary research (Rosnow & Rosenthal, 1996; Rosnow, Rosenthal, & Rubin, 2000), and have enabled authors to transform many statistical tests of significance such as *t* tests, *F* tests and *χ* ^2^ values to a metric which allows comprehension of the magnitude of the intervention effect. A very useful online calculator has also been developed, this allows authors to choose the type of raw data available, and the calculator automatically transforms this to various effect size types, including the SMD (Lipsey & Wilson, 2001).

#### 4.3.8 Unit of analysis issues

If studies involve group‐level allocation, where possible, data will be included which have been adjusted to account for the effects of clustering, typically through the use of multilevel modelling or adjusting estimates using the intra‐cluster correlation coefficient (ICC). Where the effects of clustering have not been taken into account, estimates of effect size will be adjusted following guidance in the Cochrane Handbook. If ICC is not reported external estimates will be obtained from studies that provide the best match on outcome measures and types of clusters from existing databases of ICCs (Ukoumunne, Gulliford, Chinn, Sterne, & Burney, 1999) or other similar studies within the review.

#### 4.3.9 Dealing with missing data

If study reports do not contain sufficient data to allow calculation of effect size estimates authors will be contacted to obtain necessary summary data, such as means and standard deviations or standard errors. If no information is forthcoming the study cannot be included in meta‐analysis and will instead be included in a narrative synthesis.

#### 4.3.10 Assessment of heterogeneity

Heterogeneity will be assessed first, through visual inspection of the forest plot and checking for overlap of confidence intervals and second through the *Q*, *I* ^2^ and *τ* ^2^ statistics.

#### 4.3.11 Assessment of reporting biases

A funnel plot and Egger's linear regression test will be included to check for publication bias across included studies (Sterne & Egger, 2005). Where the funnel plot is asymmetrical this indicates either publication bias or bias which relates to smaller studies showing larger treatment effects. The trim and fill method will be used where the funnel plot is asymmetrical (Higgins & Green, 2016), this is a nonparametric technique which removes the smaller studies causing irregularity until there is a new symmetrical pooled estimate, the studies which were eliminated where then filled back in to reflect the new estimate.

To ensure robustness of the review and to account for individual studies that appear to exert an undue influence on findings, process sensitivity analysis will also be carried out on domains relating to the quality of the included studies.

#### 4.3.12 Data synthesis

All analyses will be conducted using the R program. A random‐effects analysis (REM) is chosen as the hierarchical linear model. This decision to employ a REM is made for two reasons. First, the true effect would vary from study to study due to the distribution of effects. These variances may include: the setting of the intervention, the training of the person delivering the program or the dosage of the intervention. Second, under the random‐effects model the weights assigned to each individual study are more reasonable as it considers that the effect observed within each study are based on a sample from a population with an unknown mean.

Meta‐analysis will be conducted to test effectiveness of interventions to improve HSC access across various domains relating to homelessness. The outcomes related to homelessness are continuous and so the effect size metric chosen is Hedges' *g*, many studies will need to be recalculated into a standardised mean difference (SMD) with a 95% confidence interval to allow appropriate summary of effect sizes across the included studies. SMD will be calculated from means and standard deviations in the first instance, however, if a study does not provide this raw data, authors will be contacted, and this information will be requested. Failing this, many papers have been published to assist authors in calculating the SMD from primary research (Rosnow & Rosenthal, 1996; Rosnow et al., 2000) and have enabled authors to transform many statistical tests of significance such as *t* tests, *F* tests and *χ* ^2^ values to a metric which allows comprehension of the magnitude of the intervention effect. A very useful online calculator has also been developed, this allows authors to choose the type of raw data available, and the calculator will automatically transform this to various effect size types, including the SMD (Lipsey & Wilson, 2001).

#### 4.3.13 Subgroup analysis and investigation of heterogeneity

We will conduct a number of subgroup analyses to explore whether study, intervention or sample characteristics influenced the overall effect of the intervention on each outcome. The moderating variables include:

- The methodological quality of the study,
- The age of participants,
- The gender of participants,
- Type of homelessness (according to the FEANTSA classification),
- Whether the intervention was aimed at single people or families,
- How the intervention was classified (according to the framework discussed earlier) as aiming to increase access to services through improving the availability, acceptability or affordability of the programme.

#### 4.3.14 Treatment of qualitative research

The qualitative research included in this review is based upon existing evidence collated through an EGM constructed by White, Saran et al. (2018) and White, Wood, and Fitzpatrick (2018). The EGM was commissioned by the CHI and presents 292 qualitative process evaluations on the implementation issues of interventions designed to target homelessness. A process evaluation aims to examine how well the program is working and if its implementation followed the intended design. Qualitative evidence that examines the detail of how an intervention is delivered, accessed and experienced by providers and service users enable us to answer questions about why an intervention works (or does not work), who it works for and under what circumstances. This can be used to inform program and intervention development and service improvement. These were screened on 10th May 2019 for duplicates.

We will include process evaluations and other relevant qualitative studies that provide data that enables a deeper understanding of why an intervention does (or does not) work as intended, for whom and under what circumstances. We intend to describe the characteristics of included qualitative studies in terms of what qualitative methods have been used to capture this rich data, the number of interviews/focus groups/observations that have taken place, who participated and the nature of qualitative data collection (type and time taken). For example, Tinland et al. (2013) make direct observations on participants but additionally carry out in depth interviews and focus groups with policy makers and practitioners. Similarly, Luffborough (2017) carried out a mixed methods study by administering pre and posttest surveys to 108 homeless men, observing their participation in programme activities and interviewing a sample of 10 on their perceptions of the intervention. The implementation and process evaluations will be critical in this analysis, and data gathered from observations, focus groups and interviews will add an essential and unique human perspective to this review. By including an element of qualitative evidence synthesis in our review we hope to provide a more robust and rich review of the evidence base.

The categories included in the EGM describe the factors that impact upon interventions and the implementation of these across the gathered studies. These categories were developed using an iterative process and were initially based on the implementation science framework (Aarons, Hurlburt, & Horwitz Sarah, 2011). The categories were then independently piloted against process evaluations and agreement was reached by researchers in the Campbell Collaboration, Campbell UK and Ireland, and Herriot‐Watt University. The five broad categories or levels of influence agreed are contextual factors, policy makers/funders, programme managers/implementing agency, staff/case workers and recipients. The review team recognise that in the majority of interventions, more than one of the agreed categories could act as a factor that impacts positively or negatively on the effectiveness of the intervention, or both in some cases. For this reason, the review team will initially use a Framework synthesis methodology to synthesise implementation data from the EGM under the five broad categories agreed previously. The team are aware, however, that this framework may not categorise all factors sufficiently and may have to be adapted at a later stage.

Framework synthesis is an approach that originates from a process of analysing primary research data to address policy concerns. The background theoretical and empirical literature help create an understanding of the issue into an initial conceptual framework, which develops iteratively as new data are incorporated and themes are derived from the data. This process was carried out in collaboration with researchers and academics in Heriot Watt University and the Campbell Collaboration (White, Saran et al., 2018). This synthesis method presents an opportunity to use a scaffold against which findings from the different components may be brought together and organised (Carroll, Booth, & Cooper, 2011). Its flexibility captures new understanding as data are incorporated into the framework.

Framework synthesis comprises five methodological stages:

1. Familiarisation
2. Framework Selection
3. Indexing
4. Charting
5. Mapping and Interpretation

These stages are often overlapping and may be revisited throughout the process.

The first is the familiarisation stage in which a reviewer becomes familiar with current issues and ideas about the topic, by drawing iteratively on a variety of sources (Booth & Carroll, 2015). This leads to the second stage: framework selection where an initial framework is chosen, which might be a conceptual or policy framework, logic model, causal chain or established theory that might explain the issue (Brunton, Oliver, & Thomas, 2020). During the third indexing stage, studies are searched for, screened and data extracted using the initial conceptual framework. Much of this work has been carried out in the development of the Implementation issues EGM (White, Saran et al., 2018). Here, studies are sorted to determine their relevance to the review questions and to identify their main characteristics. During this stage, the Campbell UK and Ireland team will screen the process evaluations for relevance to the review. During the fourth charting stage, the main characteristics of each study will be analysed by grouping characteristics into categories and deriving themes directly from those data (Brunton et al., 2020). At this stage, a process of purposive sampling (Booth et al., 2016) will be completed by Campbell UK and Ireland. This purposive sample will endeavour to include process evaluations spanning geography, targeted populations and types of intervention in order to exhibit an accurate representation of accommodation programmes available. The selected process evaluations should present the most “rich” and “thick” data (Booth et al., 2016) from the studies included in the map. At this stage, Campbell UK and Ireland will synthesis the available data from the selected studies against the original agreed framework embedded in the EGM. During the final stage of the mapping and interpretation stage, the derived themes will be considered in light of the original research questions (Brunton et al., 2020) and in this case, policy implications. At this stage, the team will collaborate with content experts who will consider these themes in light of the available empirical and theoretical literature. In the relevant interventions available for meta‐analysis, some process evaluations of these interventions identified will be included in an additional thematic synthesis of qualitative data.

The quality of these mixed methods studies will be assessed using a tool developed by White and Keenan (2018). Along with the tool, the review team intend to use a thematic synthesis methodology to generate new themes and create meaningful relationships between these themes (Fleming, Booth, Garside, Noyes, & Tunçalp, 2019). The tool is similar to the fidelity assessment used by Stergiopoulos and Politis (2013) and aims to provide an accurate account of the eligible qualitative studies. The tool will consider methodology, recruitment and sampling, bias, ethics, analysis and findings, therefore providing a compelling justification for the inclusion of qualitative data. This tool will capture the factors that impact upon intervention effectiveness which can be viewed through the lens of all perspectives. For example, within the context of service delivery politics, policies, welfare and healthcare systems. Similarly, fidelity and implementation problems can impact upon the effectiveness of the intervention. From the perspective of the service user, who can access the services along with the barriers and facilitators of uptake will also impact on the effectiveness of the intervention. The experience that the service user receives in terms of acceptability and dropout rate will cause additional impact. All of these factors of impact along with lessons learnt by Soilemezi and Linceviciute (2018) will be carefully considered during the process of thematic synthesis.

## CONTRIBUTIONS OF AUTHORS

The review will be undertaken by systematic review specialists within the Campbell UK & Ireland Centre. S. M. will be the Principal Investigator (PI) of the project and will have overall responsibility for its conduct and delivery. S. M. will be responsible for the day to day operation of the review. This review will be supported by specialist input from C. K. and J. H. alongside research support from two full time research assistants.

- Content: A. M., P. M. and S. F.
- Systematic review methods: C. K., S. M., J. H., J. H. and C. C.
- Statistical analysis: C. K., S. M. and J. H.
- Information retrieval: C. K., S. M. and J. H.

## DECLARATIONS OF INTEREST

None to declare

### PRELIMINARY TIMEFRAME

Approximate date for submission of the systematic review: October 2020

Please note this should be no longer than two years after protocol approval. If the review is not submitted by then, the review area may be opened up for other authors.

### PLANS FOR UPDATING THIS REVIEW

We will update this review when funding is secured

## SUMMARY OF FINDINGS TABLES

**Additional tables**

1. **Data collection form for homelessness reviews**

**Table:** 

**1. Bibliographic information**	
Article ID	FREETEXT
Linked articles	FREETEXT
Extracted by	FREETEXT
Checked by	FREETEXT
Year of publication	FREETEXT
Type of publication	1. Journal Article 2. Book/book chapter 3. Government report 4. Conference proceedings 5. Presentation 6. Thesis or Dissertation 7. Unpublished report 8. Other (please specify)
Location of study	1. UK 2. ROI 3. Rest of Europe 4. USA 5. Canada 6. South America 7. Central America 8. Oceania 9. Middle‐East 10. Asia 11. Africa 12. Other (Please Specify)
*The location in which the study is set **not** where the study authors are based*.	
	Not Specified
Study funding sources	1. Research council funding 2. University scholarships and bursaries 3. Salaried research assistantships from university departments 4. Grants or loans from trusts and charities 5. Local enterprise initiatives 6. Company sponsorship 7. Government loans 8. EU Scholarships 9. Industry sponsorship 10. Other (please specify)
Possible conflicts of interest	1. Yes, possible/definite conflict of interest 2. No, study appears to be free of CoI 3. Can't tell
**2. Participant information**	
Recruitment setting	1. Clinical setting 2. Accommodation for individuals experiencing homelessness 3. Family home 4. The street 5. Community setting 6. Referred by friends or family 7. Referred by medical health professional 8. Housing Agency 9. Other (Please specify)
*Where were participants recruited from?*	
Homelessness Status at intake	1. Sleeping 'Rough' (or rooflessness) 2. Temporary Accommodation 3. Insecure Accommodation 4. Inadequate Accommodation 5. Involuntary sharing, e.g., domestic violence 6. Hidden/concealed homelessness 7. Other (please specify)
*Describe the housing status of the sample at intake and/or any information given about housing status prior to intake. Tick all that apply and try to extract numbers were available*.	
*Homelessness is defined as those individuals who are sleeping 'rough' (sometimes defined as street homeless), those in temporary accommodation (such as shelters and hostels), those in insecure accommodation (such as those facing eviction or in abusive or unsafe environments), and those in inadequate accommodation (environments which are unhygienic and/or overcrowded)*.	
	Not Specified
Geographical context	1. Urban 2. Rural 3. Suburban 4. Mixed 5. Other (please specify)
*Where participants receive treatment?*	
	Not Specified
Gender	FREETEXT
*% (actual number)*
Age	1. Under 25 2. 25 and Over
*Extract mean age, SD and range*.	
*Choose multiple options if the analysis is reported separately for different age groups*.
Complexity of needs	1. Poor Physical Health 2. Poor Mental Health 3. Incarceration 4. Substance Abuse Issues 5. Care leaver 6. Limited access to integrated support services 7. High Risk of Harm and/or Exploitation 8. Other (please specify)
*What other challenges does the individual face, if any, aside from the risk or experience of homelessness?*	
*High Risk of Harm and/or Exploitation ‐ For example, women in shelters, newcomer families, refugee/asylum seeker, care leavers*	
	Not Relevant
	Not Specified
Mental health status	1. Receiving treatment 2. Not receiving treatment 3. Other (please specify)
	Not relevant
	Not Specified
Substance use status	1. Receiving treatment 2. Not receiving treatment 3. Other (please specify)
	Not relevant
	Not Specified
Homelessness status	1. Sleeping “rough” 2. Temporary accommodation 3. Insecure accommodation 4. Inadequate accommodation 5. Other (please specify)
*Homelessness is defined as those individuals who are sleeping “rough” (sometimes defined as street homeless), those in temporary accommodation (such as shelters and hostels), those in insecure accommodation (such as those facing eviction or in abusive or unsafe environments), and those in inadequate accommodation (environments which are unhygienic and/or overcrowded)*.	
	Not Specified
Family vs. No Family	1. Family 2. Non‐Family
*Family* = *any child involved*	
*Non‐family* = *single person or couple without children*	
	Not Specified
*If mixed sample select both and describe*	
Sample size of treatment group	FREETEXT
*Number of people assigned to treatment. If more than one treatment group extract all and be clear which group is which*.	
Sample size of control group	FREETEXT
*Number of people assigned to control. If more than one control group extract all and be clear which group is which*.	
**3. Intervention information**	
How many intervention arms in this trial?	FREETEXT
*List how many study arms there are and given each a name. e.g., Intervention* = *Critical Time Intervention; Control* = *Treatment as usual*	
*If there is more than one intervention arm go to the* “*Study Arm*” *tab and add the RELEVANT study arms. You must then extract data for each relevant study arm*.	
Name of intervention	FREETEXT
*Write in the name of the program, intervention, or treatment under study. This may be specific like “critical time intervention” or it may be something more generic like “supported housing”*	
Briefly Describe the intervention	FREETEXT
*Briefly describe the intervention, what participants are offered and any important factors such as conditionality, nature of housing, case management, substance abuse treatment included etc*.	
Theory of change	FREETEXT
*How does the intervention aim to bring about change? What is the underlying theoretical rationale for why the intervention might work to improve outcomes?*	
*If not specified write* “*not specified*”	
What is the size of accommodation/How many beds?	FREETEXT
Duration of treatment period from start to finish	FREETEXT
*In the dosage items, we are interested in the amount of treatment received by the participants. If the treatment was delivered directly to participants, the authors will probably provide at least some information about dosage and you can code these items accordingly. If minimal information is provided, you should try to give estimates for these items if you can come up with a reasonable estimate*.	
Timing	1. Once a month 2. Less than weekly 3. Once a week 4. 1–2 times a week 5. times a week 6. 2–3 times a week 7. times a week 8. 3–4 times a week 9. times a week 10. Daily contact
*Frequency of contact between participants and provider/program activity*	
	Can't Estimate
Length of each individual session	FREETEXT
*How long does each contact/session last?*	
Study Personnel	1. Graduate Researcher 2. Grad/Undergrad Students 3. Author 4. Homelessness professional
*The primary individual/s who have direct contact with the participants served by the program*.	
	*Includes case manager, social worker, outreach worker*
	6. Peers 7. Interventionist (Not Hired by Researcher) 8. Interventionist (Hired by Researcher) 9. Self‐Directed 10. Medical Professionals 11. Other (please specify)
*If the report is the author's dissertation (or based on the author's dissertation), then code as* “*Graduate Researcher*”.	
*If the delivery is performed by graduate or undergraduate students assisting the author then select* “*Grad/Undergrad Students*”.	
*Code “Self‐directed” for studies where electronic/computer programs are used*.	
	Not Specified
*If the intervention is solely environmental, i.e., community housing, then code “environmental change”*	
Did provider receive specialised training?	1. Yes 2. The interventionist IS program developer 3. No
*This refers to whether or not the “interventionist” received specialised training to equip them to deliver the intervention proficiently*.	
	Not specified
Resource requirements	FREETEXT
*Time, staff, housing provision, etc*.	
Cost	FREETEXT
**4a. Study Design**	
Design	1. Randomised control trial
	*Individual or cluster randomised*
*The studies included in all reviews must include an intervention group and at least one untrained control group. Control groups can include placebo, no treatment, waitlist, or treatments vs “treatment as usual”. Any study which includes one group pretest/posttest or in which a treatment group is only compared to another treatment group will not be eligible for inclusion*.	
	2. Non‐randomised control trial
What do control subjects receive?	1. Placebo 2. Treatment as usual 3. No treatment
1. *Placebo (or attention) treatment. Group gets some attention or a sham treatment* 2. *Treatment as usual. Group gets “usual” handling instead of some special treatment*. 3. *No treatment. Group gets no treatment at all*.	
	Not specified
Unit of allocation	1. Individual 2. Group 3. Regions 4. Other (Please Specify)
*Individual (i.e., some were assigned to treatment group, some to comparison group)*	
*Group (i.e., whole subsets assigned to treatment and comparison groups)*	
*Regions (i.e., region assigned as an intact unit)*	Not Specified
Method of assignment	1. Randomly after matching 2. Randomly without matching 3. Regression discontinuity design 4. Cluster assigned 5. Wait list control 6. Non‐random, but matched 7. Other (Please Specify)
*Method of group assignment. How participants/units were assigned to groups. This item focuses on the initial method of assignment to groups, regardless of subsequent degradations due to attrition, refusal, etc. prior to treatment onset*.	
1. *Randomly after matching, yoking, stratification, blocking, etc. The entire sample is matched or blocked first, then assigned to treatment and comparison groups within pairs or blocks. This does not refer to blocking after treatment for the data analysis*. 2. *Randomly without matching, etc. This also includes cases when every other person goes to the control group*. 3. *Regression discontinuity design: quantitative cutting point defines groups on some continuum (this is rare)*. 4. *Cluster assigned, this is to be used in cluster assignment studies only, specify the number of clusters in the treatment group and the number of clusters in control*. 5. *Wait list control or other quasi‐random procedure presumed to produce comparable groups (no obvious differences). This applies to groups which have individuals apparently randomly assigned by some naturally occurring process, e.g., first person to walk in the door. The key here is that the procedure used to select groups doesn't involve individual characteristics of persons so that the groups generated should be essentially equivalent*. 6. *Non‐random, but matched: Matching refers to the process by which comparison groups are generated by identifying individuals or groups that are comparable to the treatment group using various characteristics of the treatment group. Matching can be done individually, e.g., by selecting a control subject for each intervention subject who is the same age, gender, and so forth, or on a group basis*.	
	Not Specified
Was there >20% attrition in either/both groups?	FREETEXT
*Attrition occurs when participants are lost from an intervention over time or over a series of sequential processes. Studies may describe this as “lost to follow‐up,” or “drop outs*”.	
**4b. Non‐random studies**	
How were groups matched?	1. Matched on Pretest measure 2. Matched on personal characteristics 3. Matched on demographics 4. Groups weren't matched 5. Other (please specify)
*If matching was used prior to assignment of condition, how were groups matched?*	
	Not specified
Was the equivalence of groups tested at pretest?	FREETEXT
Results of statistical comparisons of pretest differences	1. No statistically significant differences 2. Significant differences judged unimportant by coder 3. Significant differences judged of uncertain importance by coder 4. Significant differences judged important by coder 5. Other (please specify)
Were there pretest adjustments?	FREETEXT
**5.** Qualitative information	
Qualitative methods used	FREETEXT
Data analysis technique and procedure	FREETEXT
Was the intervention implemented as intended?	1. Yes 2. No
	Not specified
How was this measured?	FREETEXT
What implementation and process factors impact intervention delivery?	1. Contextual factors 2. Policy makers/funders 3. Programme managers/Implementing agency, 4. Staff/case workers 5. Recipients
**6**. Assessing quality in RCTs (Cochranes ROB2 tool)	
**Domain 1:** Risk of bias arising from the randomization process	
1.1 Was the allocation sequence random?	1. Yes 2. Probably yes 3. Probably No 4. No
1.2 Was the allocation sequence concealed until participants were enrolled and assigned to interventions?	1. Yes 2. Probably yes 3. Probably No 4. No
1.3 Did baseline differences between intervention groups suggest a problem with the randomization process?	1. Yes 2. Probably yes 3. Probably No 4. No
Risk‐of‐bias judgement	1. Low 2. High 3. Some concerns
Optional: What is the predicted direction of bias arising from the randomization process?	1. Favours experimental 2. Favours comparator 3. Towards null 4. Away from null 5. Unpredictable
**Domain 2:** Risk of bias due to deviations from the intended interventions (effect of assignment to intervention)	
2.1. Were participants aware of their assigned intervention during the trial?	1. Yes 2. Probably yes 3. Probably No 4. No
2.2. Were carers and people delivering the interventions aware of participants' assigned intervention during the trial?	1. Yes 2. Probably yes 3. Probably No 4. No
2.3. If Y/PY/NI to 2.1 or 2.2: Were there deviations from the intended intervention that arose because of the experimental context?	1. Yes 2. Probably yes 3. Probably No 4. No
2.4. If Y/PY to 2.3: Were these deviations from intended intervention balanced between groups?	1. Yes 2. Probably yes 3. Probably No 4. No
2.5 If N/PN/NI to 2.4: Were these deviations likely to have affected the outcome?	1. Yes 2. Probably yes 3. Probably No 4. No
2.6 Was an appropriate analysis used to estimate the effect of assignment to intervention?	1. Yes 2. Probably yes 3. Probably No 4. No
2.7 If N/PN/NI to 2.6: Was there potential for a substantial impact (on the result) of the failure to analyse participants in the group to which they were randomized?	1. Yes 2. Probably yes 3. Probably No 4. No
Risk‐of‐bias judgement	1. Low 2. High 3. Some concerns
Optional: What is the predicted direction of bias due to deviations from intended interventions?	1. Favours experimental 2. Favours comparator 3. Towards null 4. Away from null 5. Unpredictable
**Domain 3:** Missing outcome data	
3.1 Were data for this outcome available for all, or nearly all, participants randomized?	1. Yes 2. Probably yes 3. Probably No 4. No
3.2 If N/PN/NI to 3.1: Is there evidence that result was not biased by missing outcome data?	1. Yes 2. Probably yes 3. Probably No 4. No
3.3 If N/PN to 3.2: Could missingness in the outcome depend on its true value?	1. Yes 2. Probably yes 3. Probably No 4. No
3.4 If Y/PY/NI to 3.3: Do the proportions of missing outcome data differ between intervention groups?	1. Yes 2. Probably yes 3. Probably No 4. No
3.5 If Y/PY/NI to 3.3: Is it likely that missingness in the outcome depended on its true value?	1. Yes 2. Probably yes 3. Probably No 4. No
Risk‐of‐bias judgement	1. Low 2. High 3. Some concerns
Optional: What is the predicted direction of bias due to missing outcome data?	1. Favours experimental 2. Favours comparator 3. Towards null 4. Away from null 5. Unpredictable
**Domain 4:** Risk of bias in measurement of the outcome	
4.1 Was the method of measuring the outcome inappropriate?	1. Yes 2. Probably yes 3. Probably No 4. No
4.2 Could measurement or ascertainment of the outcome have differed between intervention groups?	1. Yes 2. Probably yes 3. Probably No 4. No
4.3 If N/PN/NI to 4.1 and 4.2: Were outcome assessors aware of the intervention received by study participants?	1. Yes 2. Probably yes 3. Probably No 4. No
4.4 If Y/PY/NI to 4.3: Could assessment of the outcome have been influenced by knowledge of intervention received?	1. Yes 2. Probably yes 3. Probably No 4. No
4.5 If Y/PY/NI to 4.4: Is it likely that assessment of the outcome was influenced by knowledge of intervention received?	1. Yes 2. Probably yes 3. Probably No 4. No
Risk‐of‐bias judgement	1. Low 2. High 3. Some concerns
Optional: What is the predicted direction of bias in measurement of the outcome?	1. Favours experimental 2. Favours comparator 3. Towards null 4. Away from null 5. Unpredictable
**Domain 5:** Risk of bias in selection of the reported result	
5.1 Was the trial analysed in accordance with a prespecified plan that was finalized before unblinded outcome data were available for analysis?	1. Yes 2. Probably yes 3. Probably No 4. No
Is the numerical result being assessed likely to have been selected, on the basis of the results, from…	
5.2…. multiple outcome measurements (e.g., scales, definitions, time points) within the outcome domain?	1. Yes 2. Probably yes 3. Probably No 4. No
5.3… multiple analyses of the data?	1. Yes 2. Probably yes 3. Probably No 4. No
Risk‐of‐bias judgement	1. Low 2. High 3. Some concerns
Optional: What is the predicted direction of bias due to selection of the reported result?	1. Favours experimental 2. Favours comparator 3. Towards null 4. Away from null 5. Unpredictable
**Overall risk of bias**	
Risk‐of‐bias judgement	1. Low 2. High 3. Some concerns
7. Assessing quality in Non‐random control trials (ROBINS‐I tool)	
**Bias due to confounding**	
1.1 Is there potential for confounding of the effect of intervention in this study?	1. Yes 2. Probably yes 3. Probably No 4. No
**If N/PN to 1.1:** the study can be considered to be at low risk of bias due to confounding and no further signalling questions need be considered	
**If Y/PY to 1.1**: determine whether there is a need to assess time‐varying confounding:	1. Yes 2. Probably yes 3. Probably No 4. No
1.2. Was the analysis based on splitting participants' follow up time according to intervention received?	1. Yes 2. Probably yes 3. Probably No 4. No
**If N/PN**, answer questions relating to baseline confounding (1.4 to 1.6)	
**If Y/PY**, go to question 1.3.	
1.3. Were intervention discontinuations or switches likely to be related to factors that are prognostic for the outcome?	1. Yes 2. Probably yes 3. Probably No 4. No
**If N/PN**, answer questions relating to baseline confounding (1.4 to 1.6)	
**If Y/PY**, answer questions relating to both baseline and time‐varying confounding (1.7 and 1.8)	
**Questions relating to baseline confounding only**	
1.4. Did the authors use an appropriate analysis method that controlled for all the important confounding domains?	1. Yes 2. Probably yes 3. Probably No 4. No
1.5. **If Y/PY to 1.4**: Were confounding domains that were controlled for measured validly and reliably by the variables available in this study?	1. Yes 2. Probably yes 3. Probably No 4. No
1.6. Did the authors control for any post‐intervention variables that could have been affected by the intervention?	1. Yes 2. Probably yes 3. Probably No 4. No
**Questions relating to baseline and time‐varying confounding**	
1.7. Did the authors use an appropriate analysis method that controlled for all the important confounding domains and for time‐varying confounding?	1. Yes 2. Probably yes 3. Probably No 4. No
1.8. If **Y/PY** to 1.7: Were confounding domains that were controlled for measured validly and reliably by the variables available in this study?	1. Yes 2. Probably yes 3. Probably No 4. No
Risk‐of‐bias judgement	1. Low 2. Moderate 3. Serious 4. Critical
Optional: What is the predicted direction of bias due to confounding?	1. Favours experimental 2. Favours comparator 3. Unpredictable
**Bias in selection of participants into the study**	
2.1. Was selection of participants into the study (or into the analysis) based on participant characteristics observed after the start of intervention?	1. Yes 2. Probably yes 3. Probably No 4. No
**If N/PN to 2.1:** go to 2.4	
2.2. **If Y/PY to 2.1**: Were the post‐intervention variables that influenced selection likely to be associated with intervention?	1. Yes 2. Probably yes 3. Probably No 4. No
2.3 **If Y/PY to 2.2**: Were the post‐intervention variables that influenced selection likely to be influenced by the outcome or a cause of the outcome?	1. Yes 2. Probably yes 3. Probably No 4. No
2.4. Do start of follow‐up and start of intervention coincide for most participants?	1. Yes 2. Probably yes 3. Probably No 4. No
2.5. **If Y/PY to 2.2 and 2.3, or N/PN to 2.4**: Were adjustment techniques used that are likely to correct for the presence of selection biases?	1. Yes 2. Probably yes 3. Probably No 4. No
Risk‐of‐bias judgement	1. Low 2. Moderate 3. Serious 4. Critical
Optional: What is the predicted direction of bias due to selection of participants into the study?	1. Favours experimental 2. Favours comparator 3. Towards null 4. Away from null 5. Unpredictable
**Bias in classification of interventions**	
3.1 Were intervention groups clearly defined?	1. Yes 2. Probably yes 3. Probably No 4. No
3.2 Was the information used to define intervention groups recorded at the start of the intervention?	1. Yes 2. Probably yes 3. Probably No 4. No
3.3 Could classification of intervention status have been affected by knowledge of the outcome or risk of the outcome?	1. Yes 2. Probably yes 3. Probably No 4. No
Risk‐of‐bias judgement	1. Low 2. Moderate 3. Serious 4. Critical
Optional: What is the predicted direction of bias due to classification of interventions?	1. Favours experimental 2. Favours comparator 3. Towards null 4. Away from null 5. Unpredictable
**Bias due to deviations from intended interventions**	
If your aim for this study is to assess the effect of assignment to intervention, answer questions 4.1 and 4.2	
4.1. Were there deviations from the intended intervention beyond what would be expected in usual practice?	1. Yes 2. Probably yes 3. Probably No 4. No
4.2. **If Y/PY to 4.1**: Were these deviations from intended intervention unbalanced between groups *and* likely to have affected the outcome?	1. Yes 2. Probably yes 3. Probably No 4. No
If your aim for this study is to assess the effect of starting and adhering to intervention, answer questions 4.3 to 4.6	
4.3. Were important co‐interventions balanced across intervention groups?	1. Yes 2. Probably yes 3. Probably No 4. No
4.4. Was the intervention implemented successfully for most participants?	1. Yes 2. Probably yes 3. Probably No 4. No
4.5. Did study participants adhere to the assigned intervention regimen?	1. Yes 2. Probably yes 3. Probably No 4. No
4.6. **If N/PN to 4.3, 4.4 or 4.5**: Was an appropriate analysis used to estimate the effect of starting and adhering to the intervention?	1. Yes 2. Probably yes 3. Probably No 4. No
Risk‐of‐bias judgement	1. Low 2. Moderate 3. Serious 4. Critical
Optional: What is the predicted direction of bias due to deviations from the intended interventions?	1. Favours experimental 2. Favours comparator 3. Towards null 4. Away from null 5. Unpredictable
**Bias due to missing data**	
5.1 Were outcome data available for all, or nearly all, participants?	1. Yes 2. Probably yes 3. Probably No 4. No
5.2 Were participants excluded due to missing data on intervention status?	1. Yes 2. Probably yes 3. Probably No 4. No
5.3 Were participants excluded due to missing data on other variables needed for the analysis?	1. Yes 2. Probably yes 3. Probably No 4. No
5.4 **If PN/N to 5.1, or Y/PY to 5.2 or 5.3**: Are the proportion of participants and reasons for missing data similar across interventions?	1. Yes 2. Probably yes 3. Probably No 4. No
5.5 **If PN/N to 5.1, or Y/PY to 5.2 or 5.3**: Is there evidence that results were robust to the presence of missing data?	1. Yes 2. Probably yes 3. Probably No 4. No
Risk of bias judgement	1. Low 2. Moderate 3. Serious 4. Critical
Optional: What is the predicted direction of bias due to missing data?	1. Favours experimental 2. Favours comparator 3. Towards null 4. Away from null 5. Unpredictable
**Bias in measurement of outcomes**	
6.1 Could the outcome measure have been influenced by knowledge of the intervention received?	1. Yes 2. Probably yes 3. Probably No 4. No
6.2 Were outcome assessors aware of the intervention received by study participants?	1. Yes 2. Probably yes 3. Probably No 4. No
6.3 Were the methods of outcome assessment comparable across intervention groups?	1. Yes 2. Probably yes 3. Probably No 4. No
6.4 Were any systematic errors in measurement of the outcome related to intervention received?	1. Yes 2. Probably yes 3. Probably No 4. No
Risk of bias judgement	1. Low 2. Moderate 3. Serious 4. Critical
Optional: What is the predicted direction of bias due to measurement of outcomes?	1. Favours experimental 2. Favours comparator 3. Towards null 4. Away from null 5. Unpredictable
**Bias in selection of the reported result**	
Is the reported effect estimate likely to be selected, on the basis of the results, from…	
7.1…. multiple outcome measurements within the outcome domain?	1. Yes 2. Probably yes 3. Probably No 4. No
7.2… multiple analyses of the intervention‐outcome relationship?	1. Yes 2. Probably yes 3. Probably No 4. No
7.3… different subgroups?	1. Yes 2. Probably yes 3. Probably No 4. No
Risk of bias judgement	1. Low 2. Moderate 3. Serious 4. Critical
Optional: What is the predicted direction of bias due to selection of the reported result?	1. Favours experimental 2. Favours comparator 3. Towards null 4. Away from null 5. Unpredictable
**Overall risk of bias**	
Risk‐of‐bias judgement	1. Low 2. Moderate 3. Serious 4. Critical
**8**. Assessing quality in Qualitative studies (White and Keenan tool)	
Are the evaluation questions clearly stated?	1. Yes 2. No
Is the qualitative methodology described?	1. Yes 2. No
Is the qualitative methodology appropriate to address the evaluation questions?	1. Yes 2. No 3. Insufficient detail
Is the recruitment or sampling strategy described?	1. Yes 2. No
Is the recruitment or sampling strategy appropriate to address the evaluation questions?	1. Yes 2. No 3. Insufficient detail
Are the researcher's own position, assumptions and possible biases outlined?	1. Yes 2. No
Have ethical considerations been sufficiently considered?	1. Yes 2. No 3. Insufficient detail
Is the data analysis approach adequately described?	1. Yes 2. No
Is the data analysis sufficiently rigorous?	1. Yes 2. No
Is there a clear statement of findings?	1. Yes 2. No
Are the research findings useful?	1. Yes 2. No

## SOURCES OF SUPPORT

### INTERNAL SOURCES

- No sources of support provided.

### EXTERNAL SOURCES

- No sources of support provided.
